# A Systematic Review of Cannibalism in Austro‐Papuan Dragons (Lacertilia: Amphibolurinae), With a New Record of Cannibalism in the Peninsula Dragon (*Ctenophorus fionni*)

**DOI:** 10.1002/ece3.74590

**Published:** 2026-11-06

**Authors:** Daniel Hoops, Peri E. Bolton

**Affiliations:** ^1^ School of Natural Sciences Macquarie University Sydney New South Wales Australia; ^2^ Department of Evolution, Ecology and Organismal Biology The Ohio State University Columbus Ohio USA

**Keywords:** agamid, Australia, diet, paedophagy, saurophagy

## Abstract

In 2012, one of us observed an adult male peninsula dragon ( *Ctenophorus fionni* ) predate a juvenile conspecific. This was noteworthy as we understood peninsula dragons to be primarily insectivorous and it prompted us to investigate cannibalism more generally using a systematic, comprehensive review of the literature. Cannibalism has never been reported in any wild, natural‐living Australo‐Papuan (i.e., Amphibolurine) agamid, except in the peninsula dragon. There are now three records of cannibalism in the peninsula dragon, all from populations of the central form. This raises the possibility that peninsula dragons, or certain populations thereof, may be unusually predisposed to cannibalism. However, the current data available are too sparse to draw firm conclusions and we encourage anyone with observations of cannibalism to publish them.

## Graphical abstract

A male “central form” peninsula dragon ( *Ctenophorus fionni* ) consumes a juvenile conspecific at Mount Wudinna, South Australia. This is the third such record in peninsula dragons. Photo by Peri E. Bolton.

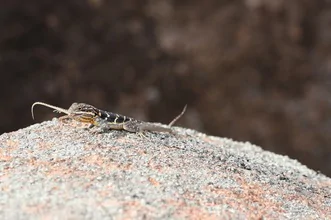

## 1 Introduction

The continent of Australia represents the epicentre of diversity for agamid lizards (Lacertilia; Agamidae) (Scarpetta et al. 2025). Agamids appear to have colonized Australia only once and subsequently diversified into a wide array of ecological niches, morphologies, and social structures (Brennan et al. 2025). The Australian agamids, all part of the subfamily Amphibolurinae, are generally diurnal and behave conspicuously, and as a result are heavily integrated into both Indigenous Australian culture and European colonial culture. For example, they are colloquially referred to as “dragons” (Hamilton et al. 2015). Globally, they are among the most recognizable reptiles due to the popularity of some species as pets.

The conspicuousness of Australian agamids has led to some species being studied as model organisms in biological research, and as such they are ecologically well‐characterized. In general, Amphibolurine agamids are primarily insectivorous with some consumption of vegetation and little to no consumption of vertebrates (Hoops 2026; Greer 1989; Cooper and Vitt 2002; Pianka 1986).

## 2 Observation

The peninsula dragon ( *Ctenophorus fionni* ) is a common and conspicuous saxicoline species of agamid lizard found on, and north of, the Eyre Peninsula in South Australia (Melville and Wilson 2019). PEB observed an adult male peninsula dragon consume a juvenile in the late afternoon on 13th January 2012 while on a recreational visit to Mt. Wudinna, an isolated outcrop of granite slabs near the town of Wudinna, South Australia (GPS: −32.9885234, 135.5477139). PEB was photographing a basking male peninsula dragon atop a small boulder (Figure 1) when it moved down the side of the boulder and then darted to chase a previously unseen juvenile at least a metre away (Figure 2; Figure S1). The adult dragon was identified as a male due to its conspicuous colouration (Melville and Wilson 2019), and the prey dragon was identified as a juvenile due to its substantially smaller size compared to the male (Figure 2). After a chase period of two minutes, the adult male started to consume the juvenile and within eight minutes of that the juvenile was completely consumed.

**FIGURE 1:**
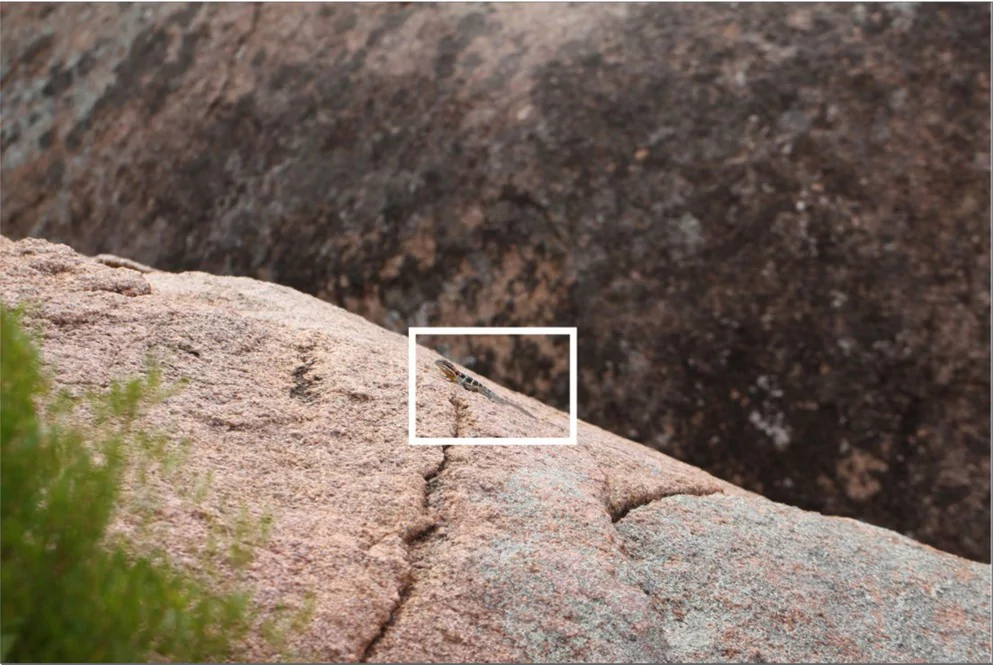
An uncropped image illustrates the context in which the observation occurred: near the top of a granite boulder at Mount Wudinna, South Australia. The adult male peninsula dragon ( *Ctenophorus fionni* ) is framed in white. Photo by Peri E. Bolton.

**FIGURE 2:**
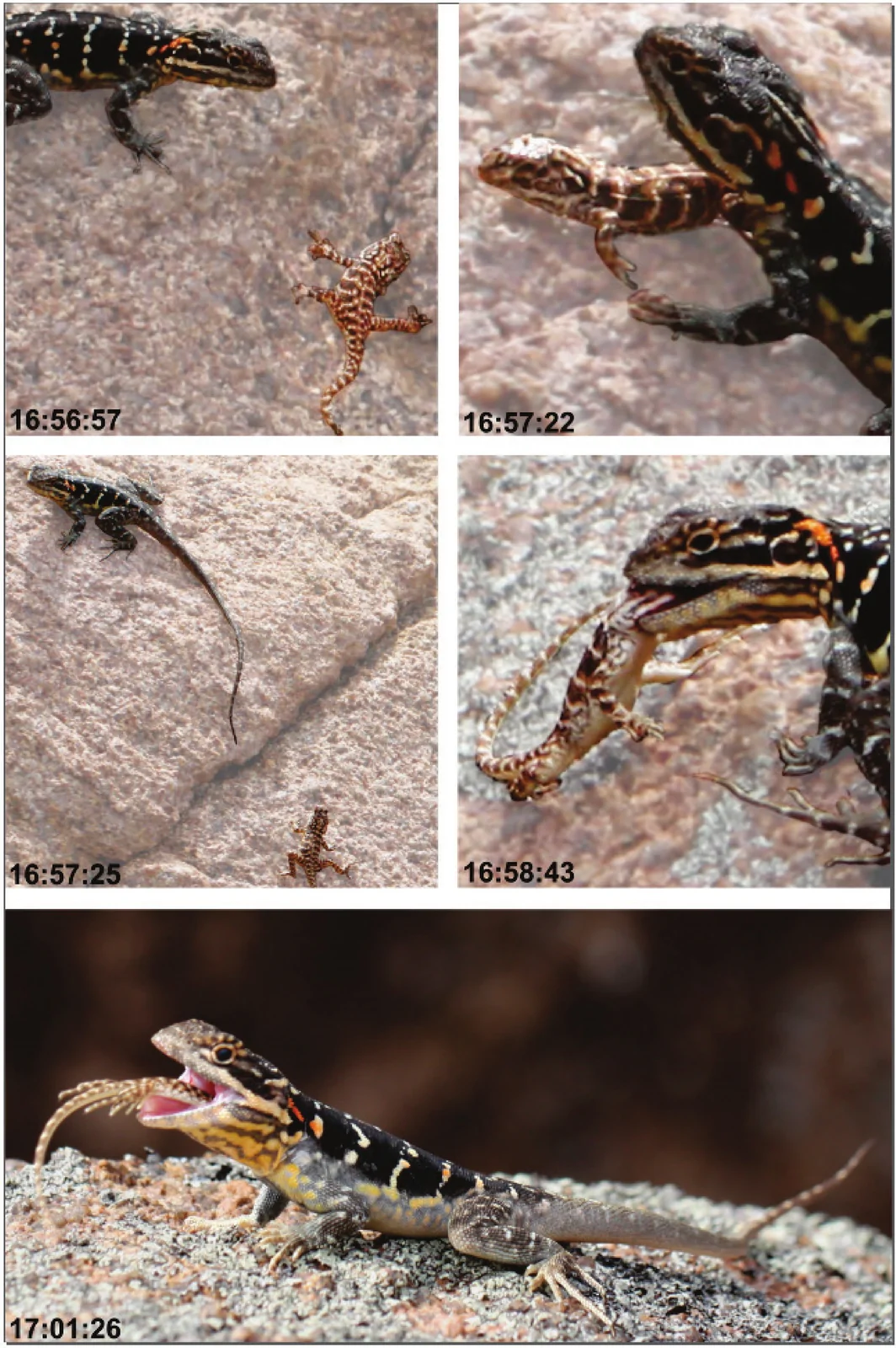
A photographic sequence, taken over five minutes, shows an adult male peninsula dragon ( *Ctenophorus fionni* ) depredating (Hanson 2006) a juvenile conspecific. The exact time each photo was taken is indicated in each lower left corner. Photos are by Peri E. Bolton and have been edited to enhance the visibility of the lizards, particularly the camouflaged juvenile, against the granite boulder. The unedited photographs are available in Figure S1.

## 3 Systematic Review Rationale

We recently conducted a comprehensive systematic review of the diets of *Ctenophorus* dragons (Hoops 2026), which revealed records of cannibalism only in peninsula dragons. Of the two records we found in the literature, one occurred in the same circumstance as our observation: an adult male consumed a juvenile (Johnston 1997, 2005). The other record is of a “piece of Peninsula Dragon skin” found in the stomach of an adult male during a diet study (Johnston 1997). The appearance of the skin, and whether it was substantial enough to determine age or sex, was not described. This observation was described as cannibalism, which we expect means the piece of skin contained both epidermal and dermal tissue, and was not semi‐transparent epidermis that would indicate the lizard had consumed naturally shed skin, which has been observed in other *Ctenophorus* (Weldon et al. 1993).^1^ These records are from Secret Rocks and Iron Duke respectively, both locations in the northern Eyre Peninsula. Therefore, all three cannibalism records (our observation and the two from the literature) refer to the “central” form of peninsula dragon (Houston 1974).

For context, two ecologically similar species, the swift dragon (*Ctenophorus modestus*) and the ornate dragon ( *Ctenophorus ornatus* ), are models in evolutionary biology (Dyke et al. 2021), with many field hours devoted to observing and recording their behaviour, and we found no records of cannibalism or attempted cannibalism in these species. One field study in the ornate dragon even seems to suggest cannibalism avoidance, reporting that “younger animals appear to be immune from the territorial harassment of adult males” (Bradshaw 1965, 54). Although this statement is made in the context of subadult dispersal due to territorial aggression, it suggests that adults avoid aggressive behaviour towards juveniles, which would presumably include predatory aggression. A similar comment in Brown (2012, 257) suggests adult tolerance towards juveniles may extend to other *Ctenophorus* species, at least in captivity. This contrasts sharply with our observation of an adult male peninsula dragon actively chasing down a juvenile over a metre away. This contrast suggests, preliminarily, that these cannibalistic events are not simply opportunistic responses by adult males to the presence of an animal of consumable size.

## 4 Systematic Review Scope and Methods

To gain a better understanding of how common cannibalism is, we conducted a review across all amphibolurine agamids, that is, all Australian and Papuan agamids. We conducted three tiers of searches. First, we conducted comprehensive, systematic searches across the scientific and “grey” literatures. Second, we searched two citizen science projects, on Facebook (www.facebook.com) and iNaturalist (www.inaturalist.org), that are devoted to collecting observations of predation events involving reptiles in Australia. Finally, we used the internet search engine Google to look for records of cannibalism in pets. Detailed methods for all our searches are available in Document S1.

## 5 Systematic Review Results

Our comprehensive, systematic search of the scientific literature returned only one additional record^2^: a subadult water dragon ( *Intellagama lesueurii* ) consumed a hatchling (Frere et al. 2015). This record was not from a natural environment, but rather from a highly urbanized setting, which can alter the diet and behaviour of wild reptiles (French et al. 2018). We could find no additional examples of cannibalism in wild, natural‐living amphibolurine agamids. This is despite numerous diet studies having been published across many species (e.g., reviewed in Greer 1989; Cooper and Vitt 2002; Hoops 2026; Abensperg‐Traun and Steven 1997; Pianka 1986).

Because of the absence of records in the scientific literature and the conspicuous nature of many agamids, we hypothesized that cannibalism events may have been observed by non‐scientists and we therefore searched two citizen science projects. Our searches returned records of predation events involving agamids (mostly insectivory), and cannibalism in other reptiles (mostly snakes). However, we did not uncover any observations of cannibalism in agamids.

Despite this lack of records in wild amphibolurine agamids, cannibalism is considered common in captivity, at least in the central bearded dragon ( *Pogona vitticeps* ) (Raiti 2012; de Vosjoli 2022; Rowland 2009; Brown 2012), one of the most popular pet lizards in the world (Valdez 2021). Brown (2012) also states that other bearded dragon species (*Pogona* sp.) and frilled dragons ( *Chlamydosaurus kingii* ) can be cannibalistic in captivity. However, our searches revealed that first‐hand accounts and verifiable evidence of cannibalism are rare^3^ (see Figure S2 for the anecdotal accounts we were able to locate). Based on our searches, we hypothesize that cannibalism is rare in pet dragons, even when the opportunity is present, but that this level of risk is still above that tolerable to pet keepers.

## 6 Discussion

Our review revealed that reports of cannibalism are exceedingly rare in Australian agamids. Our systematic, comprehensive review of the scientific literature returned observations only in the peninsula dragon and one record in urbanized water dragons. Our review of citizen science reports returned no observations of cannibalism in agamids but did return observations of agamids and observations of cannibalism, indicating the lack of records is not simply due to a difficulty in observing agamids (they are generally conspicuous) nor a lack of cannibalistic behaviour in Australian reptiles overall. Finally, our review of cannibalism in pet amphibolurines revealed that central bearded dragons ( *Pogona vitticeps* ) are commonly perceived to be cannibalistic, but that documented evidence is rare.

This overall lack of cannibalism reports stands in contrast to lizards overall. Reviews have described cannibalism as being relatively common, with numerous reports across a diversity of species including in African and Asian agamids (Mitchell 1986; Polis and Myers 1985; Ciofi et al. 2015; Oliveira et al. 2025). When cannibalism occurs in the wild, it is most likely an adult consuming a juvenile (Mitchell 1986; Polis and Myers 1985; Ciofi et al. 2015; Robbins et al. 2013). In species with sexual size dimorphism, the predator is likely to be the larger sex (Siqueira and Rocha 2008). Our observation of peninsula dragon cannibalism fits these trends: an adult male, the larger sex in this species (Houston 1974), consumed a juvenile. Therefore, though the peninsula dragon appears to be an outlier within the Amphibolurinae, the cannibalism observed in this species broadly matches the observed trends of cannibalism ecology.

In addition to the ontogenetic size difference that allows adults to consume juveniles (Ellstrand 1983), there may be specific factors that increase the probability of adults consuming juveniles. Juveniles may lack visual, olfactory, or behavioural cues that discourage cannibalism in contexts where cannibalism is a fitness disadvantage (Pfennig 1997; Rudolf 2008). Alternatively, juveniles may be the easiest targets in contexts where cannibalism provides a fitness advantage (Nishimura and Isoda 2004; Bose 2022).

The peninsula dragon may represent a novel system in which to further study the conditions that favour the evolution of cannibalism. This species is highly sexually dimorphic, with conspicuously coloured males and cryptically coloured females and juveniles (Hoops et al. 2017; Houston 1974), they likely use chemical communication through femoral pore secretions (Nafie et al. 1995), and they have exceptionally complex behavioural displays (Gibbons 1979; Wilson et al. 2021; Ramos and Peters 2021, 2015). They also occur on relatively isolated rocky outcrops, a limiting resource in a territorial species (Houston 1974; Johnston 1997). Therefore, all the factors that may favour adults consuming juveniles are likely present in the peninsula dragon.

Any adaptationist explanation of cannibalism in peninsula dragons must also contend with the absence of records in other closely related, ecologically similar Amphiboloruine species, including the swift (*Ctenophorus modestus*) and ornate ( *Ctenophorus ornatus* ) dragons referred to earlier. All the factors that may favour adults consuming juveniles are also present in these species, and we could not find any cannibalism records despite a strong literature on the ecology of both species. There is a possibility, therefore, that Australian amphibolurine agamids are an ideal comparative framework in which to examine the ecological and evolutionary factors that favour cannibalism.

Finally, we must emphasize that three records are far from sufficient to conclude that cannibalism is relatively prevalent in the central form of the peninsula dragon. Nor is the absence of records conclusive regarding the prevalence of cannibalism in other amphibolurine species. More hours of field observations, more widespread stomach content analyses, and more reporting of incidental observations by both scientists and the public are necessary before an accurate estimate of the prevalence of cannibalism across amphibolurine agamids can emerge.

## 7 Conclusion

The Australian dragons include some of the most familiar and conspicuous lizards in the world, and cannibalism appears to be rare across the subfamily. We were able to demonstrate that cannibalism is a well‐known, if rarer than currently perceived, occurrence in captive bearded dragons, and there is a single record of cannibalism in wild but urbanized water dragons. However, a comprehensive, systematic review of the published literature and additional survey of citizen science reports failed to uncover any instances of cannibalism in any wild Australian dragon lizard in a natural environment, except in the peninsula dragon ( *Ctenophorus fionni* ). Notably, tolerance of juveniles by adults has been observed in other *Ctenophorus* species.

There are now three records of cannibalism in the peninsula dragon ( *Ctenophorus fionni* ), all from populations of the “central” form. In two cases adult males consumed juveniles; in the third case an adult male consumed at least part of another dragon of unknown age and sex. Ultimately, although three observations in a single species and none in any other species is an intriguing distribution of records, there is simply not enough evidence to support any conclusions. We therefore encourage anyone with records or observations on the diets of Australian agamids, and especially anyone who has records of cannibalistic events, to report them.

## Author Contributions

**Daniel Hoops:** conceptualization (lead), data curation (lead), investigation (lead), methodology (lead), visualization (equal), writing – original draft (lead), writing – review and editing (equal). **Peri E. Bolton:** visualization (equal), writing – review and editing (equal).

## Ethics Statement

The authors have nothing to report.

## Conflicts of Interest

The authors declare no conflicts of interest.

## Supporting information


**Figure S1:** Sequential original photographs of an adult male peninsula dragon ( *Ctenophorus fionni* ) consuming a juvenile conspecific. The panels in Figure 2 have been cropped and edits from these photographs.

**Figure S2:** Anecdotal reports of cannibalism in pet bearded dragons ( *Pogona vitticeps* ).

**Document S1.** A complete description of our systematic review rationale and methods.
**Table S1:** Includes our precise search strings and returns.
**Table S2:** Includes our precise search strings and returns.

## Data Availability

A complete account of the observation, methods, and data is available in the body of the text and the [Link], [Link], [Link].

## Endnotes

^1^
This review states that captive ornate dragons (*Ctenophorus ornatus*) were observed consuming the shed skin of conspecifics. Although we do not consider this to be cannibalism, it is notable in this context.

^2^
A review of cannibalism in lizards includes an instance in the thorny devil (*Moloch horridus*) (Polis and Myers 1985, 100). However, the source they cite, Pianka and Pianka (1970), does not include a report of cannibalism in *Moloch*. Instead, we hypothesize that the inclusion of *Moloch* is based on unconfirmed reports elsewhere (Sporn 1958; Pianka et al. 1996) that neonates may sometimes, but not always (Sporn 1955; Thompson 2003), consume their eggs after hatching. This possible auto‐oophagy is beyond the scope of this review.

^3^
Although our searches did not return any observations of cannibalism in wild bearded dragons (*Pogona* sp.), we did find one case of a central bearded dragon (*Pogona vitticeps*) having consumed an adult central netted dragon (*Ctenophorus nuchalis*) (MacMillen et al. 1989).
