# Supplementary material for: A Systematic Review of Cannibalism in Austro‐Papuan Dragons (Lacertilia: Amphibolurinae), With a New Record of Cannibalism in the Peninsula Dragon (Ctenophorus fionni)
Source: Ecol Evol. 2026 Nov 6;16(11):e74590. doi: 10.1002/ece3.74590 (PMC13741859; doi:10.1002/ece3.74590)
Supplement: Supplementary file 1 [file ECE3-16-e74590-s001.docx]

**Supplementary Figure S2.** Anecdotal reports of cannibalism in pet bearded dragons (*Pogona vitticeps*).

A. A photographic record of cannibalism in pet bearded dragons. Note that the “Source” link was not functional. The original source of the photograph is unknown; it was posted to the social media platform tumblr by the user followthebluebell.

B. A still frame from a video posted on the social media platform TikTok showing a bearded dragon cannibalising the tail of a conspecific. The video is titled “Bearded Dragon Cannibal” and the username of the person who uploaded the video is unknown. Although this still frame was returned in our Google Image search and appears genuine, we cannot locate the original video on TikTok, suggesting that it may have been removed from the platform.

C. A still frame from a video posted on the social media platform Facebook showing a bearded dragon cannibalising a juvenile conspecific. The username person who uploaded the video is “RICO RICO”.

D. A still frame from a video posted on the social media platform TikTok showing two bearded dragons, one much larger than the other. Although the video does not depict an incidence of cannibalism, the accompanying narration indicates that the larger dragon started to consume the smaller dragon shortly after the image was taken. The username person who uploaded the video is “sunflowerrsee.d”.

E. A still frame from a video posted on the social media platform TikTok showing a bearded dragon cannibalising a smaller conspecific. The username person who uploaded the video is “noslews.content”.

F. A first-hand account of cannibalism in bearded dragons published on the Internet forum https://beardeddragon.org titled “My dragon is eating its 2 companions” from the user “dantethelizardsmum”. Note that the predatory dragon is stated to be a hybrid between a central bearded dragon and a Rankin’s bearded dragon (*Pogona henrylawsoni*). The species or hybrid status of the two predated dragons are unknown. URL: https://www.beardeddragon.org/threads/my-dragon-is-eating-its-2-companions.91755/

G. A first-hand account of cannibalism in bearded dragons published on the Internet forum https://ourreptileforum.com titled “The result of the content of the two bearded dragons together” from the user “dimzel”. This account is accompanied by a photograph of an apparently deceased bearded dragon with extensive injuries said to be caused by a conspecific with which it was housed.
