# Supplementary material for: A Systematic Review of Cannibalism in Austro‐Papuan Dragons (Lacertilia: Amphibolurinae), With a New Record of Cannibalism in the Peninsula Dragon (Ctenophorus fionni)
Source: Ecol Evol. 2026 Nov 6;16(11):e74590. doi: 10.1002/ece3.74590 (PMC13741859; doi:10.1002/ece3.74590)
Supplement: Supplementary file 2 [file ECE3-16-e74590-s002.docx]

**Supplementary Methods for Systematic Review**

[author redacted]

These supplementary review methods support the manuscript “**An observation of a male peninsula dragon (*Ctenophorus fionni*) consuming a juvenile, with a systematic review of cannibalism in Australian dragons (Lacertilia: Amphibolurinae)**” by [redacted].

Our systematic review methodology is based on Foo *et al.* (2021).

**1. Deciding on the review question**

*1.1 Initial question:*

Are Australian agamids cannibalistic?

*1.2 Scoping search:*

We conducted exploratory literature searches for the terms “Ctenophorus cannibalism”, “Amphibolurus cannibalism”, and “+Australia +agamid +cannibalism” (no quotes in search terms) using Google Scholar, returning 106, 116, and 135 results respectively (October 26, 2025). Searches continued iteratively as search terms were explored and refined.

*1.3 Map out the literature:*

Although we found many articles in which the diet of wild agamids is characterized either by stomach contents or by behaviour, almost none appeared to contain accounts of cannibalism. Therefore, we sought to expand our search into the grey literature and citizen science.

Furthermore, there is no phylogenetic distinction between Australian and Papuan agamids, so by searching across all Australian agamids we necessarily included Papuan agamids as well.

*1.4 Identify the question using PICO:*

Population – *Amphibolurine* agamids
Intervention/Exposure – observations of *Amphibolurine* dragons consuming conspecifics or dissection and characterization of stomach contents.
Comparator – *Amphibolurine* agamids that did not consume conspecifics despite the opportunity and capability
Outcome – reports of consumed conspecifics

**2. Executing searches**

*2.1 Literature sources:*

The primary literature sources were the two most used search engines in our experience: Web of Science and Scopus. Within Web of Science we searched 12 databases (Table S1). To supplement the primary literature sources we also searched using Google Scholar, ProQuest Dissertations & Theses, and Open Access Theses and Dissertations.

For those articles that were returned in our searches and passed the initial screening, we performed forward searches (searching the articles that cite the article in question), and backwards searches (searching all articles cited by the article in question).

We searched for citizen science records of cannibalism in amphibolurine dragons, see Section 4 for details on these searches.

We searched the Web for anecdotal reports of cannibalism in pet bearded dragons (*Pogona vitticeps*). See Section 5 for details on these searches.

*2.2 Search string generation and refinement:*

Our search string was divided into two main components.

The first component of our search string encompassed all the genera that amphibolurine dragon species have been assigned to in the peer-reviewed scientific literature. These genera were identified using the Reptile Database (Uetz *et al.* 2025), which lists all known synonyms for all currently recognized reptile species. We excluded genera that were only used before 1900, as these are genera originating from Europe and which now apply to very different European species. We also excluded genera that were coined in acts of taxonomic vandalism (Wüster *et al.* 2021) and have not subsequently received widespread acceptance (as determined by their appearance in Reptile Database synonym lists only in works of taxonomic vandalism). These are the genera that fit our criteria for inclusion:

Australo-Papuan agamid genera:

- Amphibolurus
- Chelosania
- Chlamydosaurus
- Ctenophorus
- Diporiphora
- Gowidon
- Intellagama
- Lophognathus
- Lophosaurus
- Moloch
- Pogona
- Rankinia
- Tropicagama
- Tympanocryptis

Australo-Papuan genera no longer in use:

- Physignathus
- Cryptagama
- Gemmatophora
- Caimanops
- Istiurus
- Gonocephalus
- Hypsilurus
- Gonyocephalus
- Goniocephalus

The second component of our search string was “cannibal” and its various derivatives.

*2.3 Final generalized search string:*

(Amphibolurus OR Chelosania OR Chlamydosaurus OR Ctenophorus OR Diporiphora OR Gowidon OR Intellagama OR Lophognathus OR Lophosaurus OR Moloch OR Pogona OR Rankinia OR Tropicagama OR Tympanocryptis OR Physignathus OR Cryptagama OR Gemmatophora OR Caimanops OR Istiurus OR Gonocephalus OR Hypsilurus OR Gonyocephalus OR Goniocephalus) AND (cannibal*)

*2.4 Searches:*

Table S1 includes our precise search strings and returns.

**Table S1.**

| **Search** | **Search string** |
| --- | --- |
| **Web of Science, www.webofscience.com**  2026-01-24  Indexes: Web of Science Core Collection, Chinese Science Citation Database℠, Current Contents Connect, Data Citation Index, Grants Index, KCI-Korean Journal Database, MEDLINE®, ProQuest ™ Dissertations & Theses Citation Index, SciELO Citation Index, Preprint Citation Index, Research Commons  Timespan: 1637-2026  Returns: 7 | ((((((((((((((((((((((((TS = Amphibolurus) OR (TS = chelosania)) OR (TS = chlamydosaurus)) OR (TS = Ctenophorus)) OR (TS = Diporiphora)) OR (TS = gowidon)) OR (TS = Intellagama)) OR (TS = Lophognathus)) OR (TS = Lophosaurus)) OR (TS = Moloch)) OR (TS = Pogona)) OR (TS = rankinia)) OR (TS = tropicagama)) OR (TS = Tympanocryptis)) OR (TS = Physignathus)) OR (TS = cryptagama)) OR (TS = gemmatophora)) OR (TS = caimanops)) OR (TS = istiurus)) OR (TS = Gonocephalus)) OR (TS = Hypsilurus)) OR (TS = gonyocephalus)) OR (TS = goniocephalus)) AND (TS = cannibal*)) |
| **Scopus, www.scopus.com**  2026-01-24  Timespan: all available  Returns: 1 | TITLE-ABS-KEY ( ( amphibolurus OR chelosania OR chlamydosaurus OR ctenophorus OR diporiphora OR gowidon OR intellagama OR lophognathus OR lophosaurus OR moloch OR pogona OR rankinia OR tropicagama OR tympanocryptis OR physignathus OR cryptagama OR gemmatophora OR caimanops OR istiurus OR gonocephalus OR hypsilurus OR gonyocephalus OR goniocephalus ) AND ( cannibal* ) ) |
| **ProQuest, www.proquest.com**  2026-02-01  Index: Dissertations & Theses  Timespan: all available  Returns: 13 | (Amphibolurus OR Chelosania OR Chlamydosaurus OR Ctenophorus OR Diporiphora OR Gowidon OR Intellagama OR Lophognathus OR Lophosaurus OR Moloch OR Pogona OR Rankinia OR Tropicagama OR Tympanocryptis OR Physignathus OR Cryptagama OR Gemmatophora OR Caimanops OR Istiurus OR Gonocephalus OR Hypsilurus OR Gonyocephalus OR Goniocephalus) AND (cannibal*) |
| **Open Access Theses and Dissertations**  2026-02-01  Timespan: all available  Returns: 0 | (Amphibolurus OR Chelosania OR Chlamydosaurus OR Ctenophorus OR Diporiphora OR Gowidon OR Intellagama OR Lophognathus OR Lophosaurus OR Moloch OR Pogona OR Rankinia OR Tropicagama OR Tympanocryptis OR Physignathus OR Cryptagama OR Gemmatophora OR Caimanops OR Istiurus OR Gonocephalus OR Hypsilurus OR Gonyocephalus OR Goniocephalus) AND (cannibal*) |
| **Google Scholar, scholar.google.com**  2026-01-25  Timespan: all available  Returns: “About 2680” | (Amphibolurus OR Chelosania OR Chlamydosaurus OR Ctenophorus OR Diporiphora OR Gowidon OR Intellagama OR Lophognathus OR Lophosaurus OR Moloch OR Pogona OR Rankinia OR Tropicagama OR Tympanocryptis OR Physignathus OR Cryptagama OR Gemmatophora OR Caimanops OR Istiurus OR Gonocephalus OR Hypsilurus OR Gonyocephalus OR Goniocephalus) AND (cannibal*) |

**3. Screening Search Results**

*3.1 Initial Screening:*

For each return the title, abstract, and keywords were evaluated and a return was deemed to have passed initial screening if the following criteria were met, or if the information provided was not sufficient to determine if the criteria were met. Returns that were data repositories of other returns were excluded.

Criteria:

1. The article contains empirical data
2. At least one of the study organisms is an amphibolurine agamid species
3. The article could plausibly contain an observation of cannibalism (i.e. it was not a purely genetic or morphological study)

Google Scholar returned far more articles than any other source. Because Google Scholar is much more relaxed with respect to its adherence to Boolean search terms, and due to the poor quality of its “lower down” returns (Gusenbauer and Haddaway 2020), we only screened the first 200 Google Scholar returns.

22 returns passed initial screening.

*3.2 Full-text screening:*

We read each article that passed the initial screening and extracted any reports of cannibalism described within. Two articles returned by the search engines passed the full-text screening.

*3.3 Iterative Literature Searching:*

For each article that passed initial screening we also performed iterative forwards and backwards searches. Forward searches were performed using Web of Science and returns were screened as described in Section 3. Backward searches were performed by first reading the citation list of each article and therefore were initially screened by title, followed by a screening of the abstract and keywords, followed by a full-text screening, a slight deviation from the initial screening described in Section 3.1.

One article was added to the dataset through this iterative search process.

**4. Citizen science records**

*4.1 Searches of citizen science databases.*

To look for citizen science observations we examined records on the social media platforms Facebook (www.facebook.com) and iNaturalist ([www.inaturalist.org](https://www.inaturalist.org/)).

In Facebook we searched for “cannibal” and “dragon” (two separate searches) in the Facebook group “Predation Records of Australian Reptiles and Frogs” (https://www.facebook.com/groups/1552327185231775). These searches can be replicated here: <https://www.facebook.com/groups/1552327185231775/search/?q=dragon> and [https://www.facebook.com/groups/1552327185231775/search/?q=cannibal](https://www.facebook.com/groups/1552327185231775/search/?q=cannibal.).

In iNaturalist we searched for “Dragons Family Agamidae” in the project “Australian Reptile Predation Record”, project #214567, <https://www.inaturalist.org/projects/australian-reptile-predation-record>. The search, filtering for records included in project #214567 and taxon #31096, can be replicated here: <https://www.inaturalist.org/observations?project_id=214567&taxon_id=31096&verifiable=any>.

*4.2 Search returns*

Table S2 includes our precise search strings and returns.

**Table S2.**

| **Search** | **Search string** |
| --- | --- |
| **Facebook, www.facebook.com**  2026-02-02  Group: Predators and Prey - Predation Records of Australian Reptiles and Frogs, www.facebook.com/groups/1552327185231775  Timespan: 2022-11-17 to 2026-02-02  Filters: none  Returns: 6 | cannibal |
| **Facebook, www.facebook.com**  2026-02-02  Group: Predators and Prey - Predation Records of Australian Reptiles and Frogs, www.facebook.com/groups/1552327185231775  Timespan: 2022-11-17 to 2026-02-02  Filters: none  Returns: 38 | dragon |
| **iNaturalist, www.inaturalist.org**  2026-02-02  Project: Australian Reptile Predation Record, https://inaturalist.ala.org.au/projects/australian-reptile-predation-record  Timespan: 2024-08-23 to 2026-02-02  Filters: project #214567 and taxon #31096  Returns: 17 | Dragons Family Agamidae |

*4.3 Screening citizen science returns:*

Returns were all photographic, and so were screened visually to identify the predator and the prey to determine whether both were the same species of agamid lizard. These searches did not produce any observations of cannibalism in dragons.

**5. Searches for reports of cannibalism in pet bearded dragons**

*5.1 Searches of the web for anecdotal reports of cannibalism in pet bearded dragons*

Our systematic literature searches uncovered several articles stating that cannibalism is common in pet bearded dragons (*Pogona vitticeps*) and also occurs in other bearded dragons *(Pogona* sp.) and frilled dragons *(Chlamydosaurus kingii*)*,* however these statements were made as generalizations without any substantiating evidence or citations to substantiating evidence. Furthermore, our systematic searches did not return any first-hand accounts or direct observations supporting these statements in the published literature, grey literature, or citizen science projects. We then turned to pet-keeping internet forums, message boards, and online communities to try to find any direct evidence of cannibalism in pet bearded dragons. This was initiated by searching “bearded dragon cannibal”, “frilled dragon cannibal”, and “frilled lizard cannibal” in Google Web and Google Image search engines.

*5.2 Screening pet-keeping internet forums, message boards, and online communities:*

We screened the first ten pages of Google Web returns to our searches and scrolled Google Image returns for the same search string until we reach the end of the returns. These searches uncovered six first-hand observations of cannibalism in pet bearded dragons. These six observations are included as Supplementary Figure 2.

One further observation was provided by a colleague who knew of our interest in this topic. It is also included in Supplementary Figure 2.

**References**

Daly B. G., Dickman C. R. & Crowther M. S. (2008) Causes of habitat divergence in two species of agamid lizards in arid central Australia. *Ecology* **89** , 65–76.

Foo Y. Z., O’Dea R. E., Koricheva J., Nakagawa S. & Lagisz M. (2021) A practical guide to question formation, systematic searching and study screening for literature reviews in ecology and evolution. *Methods Ecol. Evol.* **12** , 1705–1720.

Gusenbauer M. & Haddaway N. R. (2020) Which academic search systems are suitable for systematic reviews or meta‐analyses? Evaluating retrieval qualities of Google Scholar, PubMed, and 26 other resources. *Res. Synth. Methods* **11** , 181–217.

Murray P. J. (1980) *The small vertebrate community at Badgingarra, Western Australia*. Murdoch University.

O’Dea R. E., Lagisz M., Jennions M. D. *et al.* (2021) Preferred reporting items for systematic reviews and meta‐analyses in ecology and evolutionary biology: a PRISMA extension. *Biol. Rev.* **96** , 1695–1722.

Uetz P., Freed P., Aguilar R., Reyes F., Kudera J. & Hošek J. (2025) The Reptile Database. [online]. Available from: reptile-database.org [Accessed 2026].

Wüster W., Thomson S. A., O’Shea M. & Kaiser H. (2021) Confronting taxonomic vandalism in biology: conscientious community self-organization can preserve nomenclatural stability. *Biol. J. Linn. Soc.* **133** , 645–670.
