# Supplementary material for: A Systematic Review of Cannibalism in Austro‐Papuan Dragons (Lacertilia: Amphibolurinae), With a New Record of Cannibalism in the Peninsula Dragon (Ctenophorus fionni)
Source: Ecol Evol. 2026 Nov 6;16(11):e74590. doi: 10.1002/ece3.74590 (PMC13741859; doi:10.1002/ece3.74590)
Supplement: Supplementary file 3 [file ECE3-16-e74590-s003.docx]

**Supplementary Figure S1.** Sequential original photographs of an adult male peninsula dragon (*Ctenophorus fionni*) consuming a juvenile conspecific. The panels in Figure 2 have been cropped and edits from these photographs.
